# *Ex vivo* Detection and Characterization of Hepatitis B Virus-Specific CD8^+^ T Cells in Patients Considered Immune Tolerant

**DOI:** 10.3389/fimmu.2019.01319

**Published:** 2019-06-06

**Authors:** Pil Soo Sung, Dong Jun Park, Jung-Hee Kim, Ji Won Han, Eun Byul Lee, Gil Won Lee, Hee Chul Nam, Jeong Won Jang, Si Hyun Bae, Jong Young Choi, Eui-Cheol Shin, Su-Hyung Park, Seung Kew Yoon

**Affiliations:** ^1^Department of Internal Medicine, College of Medicine, Eunpyeong St. Mary's Hospital, The Catholic University of Korea, Seoul, South Korea; ^2^Department of Biomedicine & Health Sciences, The Catholic University Liver Research Center, College of Medicine, The Catholic University of Korea, Seoul, South Korea; ^3^Graduate School of Medical Science and Engineering, KAIST, Daejeon, South Korea; ^4^Department of Internal Medicine, College of Medicine, Seoul St. Mary's Hospital, The Catholic University of Korea, Seoul, South Korea

**Keywords:** hepatitis B virus, CD8^+^ T-cell response, programmed death protein-1, chronic infection, interferon-γ

## Abstract

In this study, we aimed to detect and characterize *ex vivo* virus-specific CD8^+^ T cells in patients with immune-tolerant hepatitis B virus (HBV) infection. We investigated a Korean chronic hepatitis B cohort composed of 15 patients in the immune-tolerant phase, 17 in the immune-active phase, and 13 under antiviral treatment. We performed enzyme-linked immunospot (ELISpot) assays *ex vivo* and intracellular cytokine staining after *in vitro* culture. We also performed *ex vivo* multimer staining assays and examined the expression of programmed death-1 (PD-1) and CD127 in pentamer-positive cells. *Ex vivo* ELISpot revealed that HBV-specific T cell function was weaker in immune-tolerant patients than in those under antiviral treatment. *In vitro* culture of peripheral blood mononuclear cells for 10 days revealed that HBV-specific CD8^+^ T cells produced interferon-γ in some immune-tolerant patients. We detected HBV-specific CD8^+^ T cells *ex vivo* (using the HBV core_18−27_ pentamer) in patients from all three groups. The PD-1^+^ subset of pentamer^+^ CD8^+^ T cells was smaller *ex vivo* in the immune-tolerant phase than in the immune-active phase or under antiviral treatment. Interestingly, the proportion of PD-1^+^ CD8^+^ T cells in HBV-specific CD8^+^ T cells correlated with patient age when all enrolled patients were analyzed. Overall, HBV-specific CD8^+^ T cells are present in patients considered as immune-tolerant, although their *ex vivo* functionality is significantly weaker than that in patients under antiviral treatment (*P* < 0.05). Despite the high viral load, the proportion of PD-1 expression in HBV-specific CD8^+^ T cells is lower in the immune-tolerant phase than in other phases. Our results indicate appropriate stimulation may enhance the effector function of HBV-specific CD8^+^ T cells in patients considered as being in the immune-tolerant phase.

## Introduction

Chronic hepatitis B (CHB) is a life-threatening liver disease affecting 257 million individuals worldwide, particularly in the Asia-Pacific region (1). In endemic areas, hepatitis B virus (HBV) is typically transmitted from chronically infected mothers to neonates (2). Perinatal HBV infection causes chronic infection in more than 90% of exposed individuals (3). With perinatal infection, lifetime mortality risk due to complications of liver cirrhosis (LC) or hepatocellular carcinoma (HCC) reaches 40% in men and 15% in women (3).

Traditionally, chronic HBV infection by vertical transmission is known to have several phases (4). Initially, most children with perinatal HBV infection are asymptomatic, which is traditionally referred to as the “immune-tolerant (IT)” phase and characterized by the presence of hepatitis B surface antigen (HBsAg), hepatitis B envelope antigen (HBeAg), and high serum HBV DNA levels with minimal liver inflammation. This phase was thought to persist for decades typically followed by the “immune-clearance” phase, which is characterized by elevated liver enzymes, declining HBV DNA, and spontaneous HBeAg seroconversion. The immune-clearance phase is followed by the “low-replicative” phase, with minimal liver inflammation. In this phase, up to 30% of patients have been reported to undergo viral reactivation with increased HBV DNA and liver enzymes. These HBeAg-negative patients with spontaneous reactivation show increased risks of LC and HCC, whereas the risk of fatal diseases for those who remain inactive is much lower (2, 3). However, this concept of immune tolerance is not generally accepted by recent guidelines from Europe (5).

Although perinatal transmission of HBV is considered to lead to chronic persistent infection, the underlying mechanism remains unclear. Until recently, HBV-infected children in the IT phase were considered to have defects in mounting effective humoral and T cell responses against the infecting virus (6, 7). Very weak type-I interferon (IFN) responses (8–10), robust immunosuppressive IL-10 induction (11), and impaired IL-21 secretion from follicular helper T cells (12) following HBV infection have been suggested to limit the induction of effective adaptive immune responses in patients in the IT phase.

Recently, however, the concept of immune tolerance in HBV-infected neonates has been challenged. Studies reported that HBV infection in younger patients was not associated with an immune profile of T-cell tolerance (13, 14). One of these studies showed that HBV-specific T cell responses in the IT phase were comparable to those in the immune-active (IA) phase (13). Another study revealed HBV DNA integration and clonal hepatocyte expansion in patients considered IT phase at a high rate (14). The authors suggested that clonal hepatocyte expansion resulted in a response to hepatocyte turnover mediated by HBV-specific T cells, which were detected in patients considered as immune-tolerant (14, 15). In agreement with these reports, a recent study showed that antiviral therapy in patients with HBeAg-positive CHB with a high viral load and alanine transaminase (ALT) level below normal reduced the risk of HCC (16). It has also been reported that substantial fibrosis and necroinflammatory activity already existed in the liver biopsy of some patients in the IT phase (17, 18). Therefore, recent European guidelines referred to the traditional “immune tolerant” phase as HBeAg-positive chronic HBV infection (5).

In Korea and China, genotype C HBV prevails among chronic carriers of the virus, regardless of the clinical stage of liver disease (19, 20). In general, genotype C HBV infection is associated with more severe liver disease and an increased risk of HCC (20–22). Moreover, genotype C HBV is associated with lower rates of HBeAg and/or HBsAg loss than genotypes A and B (22). However, HBV-specific T cell responses in patients with genotype C HBV infection have not been explained in detail.

In a previous study by Shin et al. (23), a correlation between HCV-specific CD8 T-cell responses in the blood and molecular and functional markers of T-cell responses in the liver was demonstrated. Thus, HCV-specific CD8 T-cell responses in the blood were valid markers of intrahepatic T-cell activity. For different phases of HBV infection, another study (24) demonstrated CD8^+^ T cell dysfunction using patients' blood samples from different infection stages. A more recent report revealed an association between blood transcriptomes and liver biopsy transcriptomes at different infection stages (25). Therefore, we used peripheral blood samples to analyze *ex vivo* T cell response in patients with different phases of HBV infection.

In this study, we performed *ex vivo* functional assays and multimer staining to investigate the existence and function of HBV-specific CD8^+^ T cells in Korean patients with CHB. We also examined the expression levels of exhaustion (PD-1) and memory marker (CD127) in multimer^+^ cells in peripheral blood samples from these patients. Although their *ex vivo* function was impaired, we confirmed the presence of HBV-specific CD8^+^ T cells containing a smaller proportion of PD-1^+^ cells in IT patients. Our results indicate that HBV-specific CD8^+^ T cells in Korean IT patients may not be tolerant or exhausted, and appropriate stimulation can enhance the effector function of HBV-specific CD8^+^ T cells in patients considered as being in the IT phase.

## Materials and Methods

### Patient Cohort and Sample Preparation

We recruited a cohort of 45 patients with CHB with human leukocyte antigen A2 (HLA-A2) alleles from Seoul St. Mary‘s hospital. Table 1 summarizes the characteristics and laboratory findings of the cohort. Forty-four patients were categorized into three different CHB phases by serum ALT levels and serologic parameters, including HBsAg, HBeAg, anti-HBeAg, and serum copies of viral DNA (4). We adopted the traditional definitions of IT and IA phases from the American Association for the Study of Liver Diseases guidelines (4). Patients in the IT group (n = 15) had normal ALT levels (< 40 IU/mL), HBeAg positivity, and consistently high HBV DNA levels (median HBV DNA = 9.09 log copies/mL) for at least 2 years. Patients in the IA group (n = 17) had elevated ALT levels. We did not divide the patients in the IA group according to HBeAg positivity. We also included patients on antiviral treatment (AT) (*n* = 13) in our cohort. Among them, 8 patients were taking entecavir (Table 1). The mean duration of antiviral treatment in patients on AT was 61.1 ± 43.5 weeks (mean ± standard deviation). Blood was also obtained from age-matched non-HBV-infected adult healthy controls (*n* = 4).

**Table 1:** Clinical parameters of study patients.

**Variables**	**IT (*n* = 15)**	**IA (*n* = 17)**	**AT (*n* = 13)**	***P*-value^**a**^ (IT vs. AT)**
Age (years), median (range)	37 (25–53)	42 (25–67)	47 (25–74)	0.005
Male sex, *n* (%)	8 (53)	11 (65)	8 (62)	
HBeAg/HBeAb				
+/–	15 (100)	11 (65)	1 (8)	
+/+	0 (0)	0 (0)	0 (0)	
–/+	0 (0)	6 (35)	9 (69)	
Median HBV DNA level (log copies/mL)	9.09	8.59	2.01	<0.001
ALT (U/L), median (range)	34 (14–38)	189 (52–1,599)	25 (15–43)	0.016
Antiviral therapy, *n* (%)				
None	15 (100)	17 (100)	0 (0)	
Adefovir	0 (0)	0 (0)	1 (8)	
Entecavir	0 (0)	0 (0)	8 (62)	
Tenofovir	0 (0)	0 (0)	4 (30)	

*ALT, Alanine aminotransferase; AT, antiviral treatment; HBeAb, hepatitis B envelope antibody; HBeAg, hepatitis B envelope antigen; HBsAg, hepatitis B surface antigen; HBV, hepatitis B virus; IA, immune-active; IT, immune-tolerant*.

a *P-value estimated by Mann-Whitney U-test or Kruskal-Wallis test*.

HBV DNA levels in serum samples were quantified using real-time PCR as previously described (7). Patients' sera were tested for HBsAg, HBeAg, and anti-HBeAg. HBV genotype was not assessed because previous reports have shown that most patients with CHB in Korea are infected with HBV genotype C (26). Peripheral blood mononuclear cells (PBMCs) were isolated using Ficoll–Hypaque density gradient centrifugation and cryopreserved for immunologic analysis. Informed consent in writing was obtained from all patients. The present study was conducted according to the Declaration of Helsinki principles and was approved by the Institutional Review Boards (Seoul St. Mary's Hospital, KC16MISI0714).

### Virus Sequencing

The genomic region covering the HBV core gene was amplified and sequenced from patients enrolled in this study. Viral DNA was extracted with the QIAamp MiniElute Virus Spin Kit (Qiagen, Hilden, Germany) according to the manufacturer's protocol. A 700-bp core fragment was amplified in a two-step nested polymerase chain reaction (PCR) using HBV-specific primers HBV core-forward (tgtcaacgaccgaccttgagg), HBV core-reverse (tgtagctcttgttcccaa), HBV core internal-forward (aggctgtaggcataaattggt), and HBV core internal-reverse (ttcccaccttatgagtccaag), as previously described (Supplementary Table 1) (27). PCR products were directly sequenced and aligned by Cosmogenetech (Seoul, Republic of Korea).

### Flow Cytometry

The following commercially available antibodies were used for multi-color flow cytometry: BV421-conjugated anti-PD-1, BV521-conjugated anti-CD3, BV605-conjugated anti-CD4, BV786-conjugated anti-chemokine receptor 7 (CCR7), APC/Cy7-conjugated anti-mouse CD4, APC-conjugated anti-mouse PD-1 (Biolegend, San Diego, CA, USA), FITC-conjugated anti-HLA-A2, FITC-conjugated anti-CD45RA, PE-TR-conjugated anti-CD14, CD19, PE-Cy7-conjugated anti-CD127, APC-H7-conjugated anti-CD8, PE-conjugated anti-IFN**-γ**, PE-Cy7-conjugated anti-tumor necrosis factor α (TNF-α), PE-conjugated anti-major histocompatibility complex (MHC)-pentamer (Proimmune, Oxford, UK), Dead cells were excluded using the LIVE/DEAD red fluorescent reactive dye (Invitrogen, Carlsbad, CA, USA). Multi-color flow cytometry was performed using the LSRII instrument (BD Biosciences), and data were analyzed using FlowJo software (TreeStar, Ashland, OR, USA). HLA-A^*^02 pentamers corresponding to HBV core_18−27_ FLPSDFFPSV, HBV core_18−27_ FLPSDFFPSI, and HBV polymerase_455−463_ were made by Proimmune. For detection of antigen-specific CD8^+^ T cells, PBMCs were incubated with pentamer for 30 min in the dark at 4 degrees Celsius and subsequently phenotyped. HLA class I genotyping was performed by flow cytometry using the anti-HLA-A2 monoclonal antibody.

### Direct *ex vivo* IFN-γ Enzyme-Linked Immunospot (ELISpot) Assay

Duplicate cultures of 300,000 PBMCs/well were set up in ELISpot plates. HLA-A2 PBMCs were stimulated with a peptide mixture (ProMix HBV Peptide Pool, Proimmune, England) at a final concentration of 1 μg/mL for 24 h (28). The sequences of HLA-A2 restricted HBV peptides are presented in Supplementary Table 2. ELISPOT assays using overlapping peptides (OLPs) of HBV core and surface proteins were carried out as previously described (7) with minor modifications. All the peptides used in our study have the sequence of HBV genotype C. After this incubation, biotinylated anti- IFN-γ detection antibody was added and streptavidin-horseradish peroxidase was used for the detection of the spots. The number of peptide-specific, IFN-γ-secreting cells was calculated by subtracting the non-stimulated control value from the stimulated sample. Positive controls were made up of cells stimulated with phytohemagglutinin (10 μg/mL). For comparison, PBMCs were also stimulated with OLPs from cytomegalovirus (CMV) pp65 (JPT, Berlin, Germany). Wells were considered positive when the spot-forming unit (SFU) was above 7 and at least 1.5 times the mean of the unstimulated control wells.

### *In vitro* Expansion of HBV-Specific T Cells

HBV-specific T cells were cultured as follows: 5 × 10^5^ PBMCs were stimulated with the HBV peptide mixture in the presence of 20 IU of IL-2 in RPMI containing 10% fetal bovine serum for 10 days. The final concentration of each peptide was 1 μg/mL. IL-2 and medium were refreshed on day 4 and 8 of culture. On day 10, intracellular cytokine staining was performed. For intracellular cytokine staining, brefeldin A (BD Biosciences) and monensin (BD Biosciences) were added, and PBMCs were stained with surface markers after 7 h of incubation. Surface marker-stained cells were permeabilized using a Foxp3 Staining Buffer Kit (eBioscience) and further stained for intracellular cytokines or transcription factors for 30 min at 4 degrees Celsius.

### *Ex vivo* Cytokine Secretion Assay

The cytokine secretion assay on pentamer^+^ CD8^+^ T cells was performed as described previously (29). Briefly, PBMCs were coupled with capture reagents (Cytokine Secretion Assay kit, Miltenyi Biotec, Auburn, AL, USA) for human IFN-γ and TNF-α under stimulation from the peptide mixture. The cells were incubated in a closed tube for 45 min at 37 degrees Celsius under slow continuous rotation using the MACS mix (Miltenyi Biotec). After a washing step, cells were resuspended in cold medium containing IFN-γ and TNF-α detection antibodies. Subsequently, the cells were resuspended in MACS buffer containing antibodies specific for surface markers, including pentamer, and flow cytometry was performed on a BD LSR II cytometer.

### Statistical Analysis

SPSS version 20 software (IBM Corp., Armonk, NY, USA) was used for statistical analyses. The discrete variables were compared using the χ^2^ test, and an independent *t*-tests were used for continuous variables. Pearson correlation tests were performed to analyze correlations between two parameters. Statistical significance was defined as a *P* < 0.05.

## Results

### Proportion of HBV-Specific CD8^+^ T Cells in IT Phase Does Not Differ From Those in Other Clinical Phases of CHB

**Figure 1:**
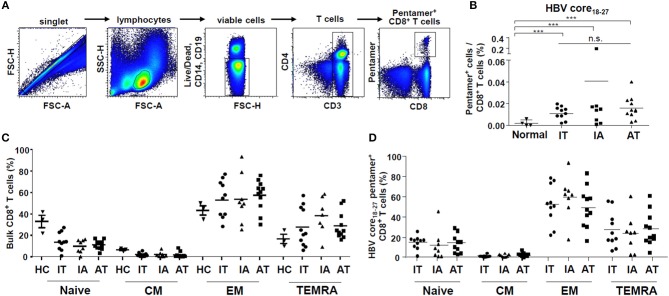
Proportion of *ex vivo* HBV core_18−27_-specific CD8^+^ T cells in the patient cohort. **(A)** Gating strategy for quantification of HBV core_18−27_-specific CD8^+^ T cells using flow cytometry. **(B)** Proportion of HBV core_18−27_-specific CD8^+^ T cells as determined using flow cytometry in normal controls and patients in the IT, IA, and AT groups (normal: *n* = 4, IT: *n* = 10, IA: *n* = 8, AT: *n* = 11). ***P < 0.001. **(C,D)** Proportions of naïve (CCR7^+^CD45RA^+^), central memory (CCR7^+^CD45RA^−^), effector memory (CCR7^−^CD45RA^−^), and effector memory RA (CCR7^−^CD45RA^+^) T cells in bulk and HBV core_18−27_-specific CD8^+^ T cells from the IT, IA, and AT groups. AT, antiviral treatment; CCR7, chemokine receptor 7; CM, central memory; EM, effector memory; HBV, hepatitis B virus; HC, healthy control; IA, immune-active; IT, immune-tolerant; TEMRA, effector memory RA T cell.

Initially, we examined the *ex vivo* proportion of HBV-specific CD8^+^ T cells in the peripheral blood from normal controls and patients in the IT, IA, and AT groups based on MHC class I multimer staining (Figure 1A). Because of limited sample availability, we performed multimer staining on 4 samples from normal controls, 10 samples from patients in the IT group, 8 samples from patients in the IA group, and 11 samples from patients under AT.

We used the HLA-A^*^02 pentamer corresponding to the HBV core_18−27_ (FLPSDFFPSV) to identify HBV-specific CD8^+^ T cells (30, 31). This pentamer can be used to detect HBV-specific CD8^+^ T cells, as it recognizes the T cell receptors of T cells induced by both the HBV core_18−27_ region/ HLA-A^*^02 complex: FLPSDFFPSI/ HLA-A^*^02 and FLPSDFFPSV/ HLA-A^*^02 (32) (Supplementary Figure 1). The substitution at position 27 I to V is known to affect peptide binding to HLA-A^*^02 molecules and does not likely affect T cell receptor recognition (31). Before multimer staining, we sequenced the corresponding epitope regions in viral DNA using sera from randomly selected patients (Supplementary Table 3). We confirmed that the sequence of the HBV core_18−27_ was FLPSDFFPSI in every sample tested (Supplementary Table 3). We compared the frequency of CD8^+^ T cells recognizing the HLA-A^*^02 pentamer to HBV core_18−27_ FLPSDFFPSV and to HBV core_18−27_ FLPSDFFPSI (Supplementary Figure 1). There were no significant differences in the frequencies of pentamer^+^ CD8^+^ T cells in our cohort when either FLPSDFFPSV/HLA-A^*^02 pentamer or FLPSDFFPSI/HLA-A^*^02 pentamer was used.

The gating strategy used to detect CD8^+^ T cells using the HBV core_18−27_ pentamer is presented in Figure 1A. As shown in Figure 1B, the proportion of HBV core_18−27_-specific CD8^+^ T cells in the IT phase did not differ from those in the other clinical phases of CHB, although the values were significantly higher than those in normal controls (*P* < 0.001) (Figure 1B and Supplementary Figure 2). When using a HBV polymerase_455−463_ pentamer, we certainly detected pentamer^+^CD8^+^ T cells in two patients with acute HBV infection and one patient in IA phase (Supplementary Figure 3). However, for chronic HBV infection with IT and AT phases, pentamer^+^CD8^+^ T cells were not readily detected when using polymerase_455−463_ pentamer, which agrees with the results of a recent study (33).

Next, we identified subsets of bulk and HBV core_18−27_-specific CD8^+^ T cells based on the expression of CCR7 and CD45RA. The proportions of naïve (CCR7^−^CD45RA^+^), central memory (CCR7^+^CD45RA^−^), effector memory (CCR7^−^CD45RA^−^), and effector memory RA (CCR7^−^CD45RA^+^) T cells in bulk and HBV core_18−27_-specific CD8^+^ T cells were calculated (Supplementary Figure 4). A recent study demonstrated that most HBV core_18−27_-specific CD8^+^ T cells are effector memory T cells regardless of the infection stage, although the proportion significantly varies among individuals (32). We found similar results, with the effector memory T cell subset showing the highest values among IT, IA, and AT groups (Figure 1D). However, there were no significant differences in the proportions of naïve, central memory, effector memory, and effector memory RA subsets in bulk CD8^+^ T cells and HBV core_18−27_-specific CD8^+^ T cells among the IT, IA, and AT groups (Figures 1C,D).

### *Ex vivo* HBV-Specific CD8^+^ T Cells in the IT Phase Have Low Proportion of PD-1^+^ Cells

**Figure 2:**
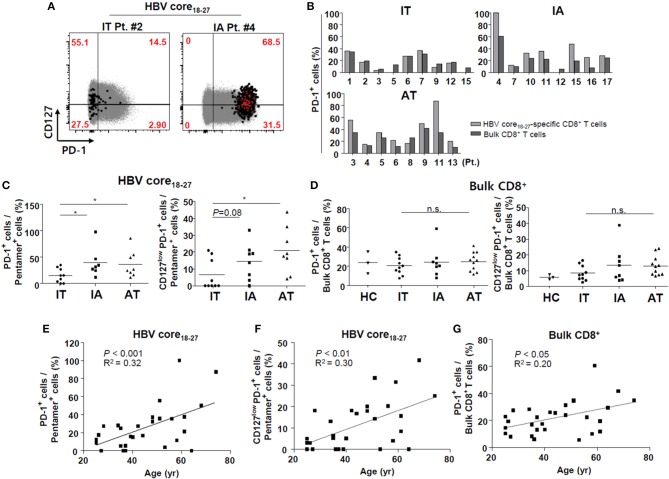
Low proportion of PD-1^+^ cells *ex vivo* in HBV core_18−27_-specific CD8^+^ T cells in the IT phase. **(A)** Representative dotplot describing the proportion of CD127^high^ and PD-1^+^ cells in total CD8^+^ T cells and HBV-specific CD8^+^ T cells. Gray-colored spots represent bulk CD8^+^ T cells and red-colored spots represent HBV-specific CD8^+^ T cells. **(B)** Proportion of PD-1^+^ cells in bulk and HBVcore_18−27_-specific CD8^+^ T cells in each patient. **(C)** Proportion of PD-1^+^/CD127^low^PD-1^+^ cells in HBVcore_18−27_-specific CD8^+^ T cells in the three groups. *P < 0.05. **(D)** Proportion of PD-1^+^/CD127^low^PD-1^+^ cells in bulk CD8^+^ T cells in the three groups. **(E–G)** Associations between age and PD-1^+^ HBV core_18−27_-specific CD8^+^ T cells (E), CD127^low^PD-1^+^ HBV-specific CD8^+^ T cells **(F)**, and PD-1^+^ bulk CD8^+^ T cells **(G)** are presented. Pearson correlation analyses were performed. AT, antiviral treatment; HBV, hepatitis B virus; HC, healthy control; IA, immune-active; IT, immune-tolerant; n.s., not significant; PD-1, programmed cell death protein-1.

Next, we investigated the surface markers of *ex vivo* HBV-specific CD8^+^ T cells in PBMCs. First, we focused on the surface expression of PD-1, a well-known T cell exhaustion marker, in bulk and HBV-specific CD8^+^ T cells. In patients in the IT group, the proportion of PD-1^+^ cells was similar between HBV core_18−27_-specific CD8^+^ T cells and bulk CD8^+^ T cells, although the proportion of PD-1^+^ cells was generally higher in HBV-specific CD8^+^ T cells than in bulk CD8^+^ T cells in the IA and AT phases (Figures 2A,B).We found that the proportion of PD-1^+^ HBV core_18−27_-specific CD8^+^ T cells was significantly lower in patients in the IT phase than in those in the IA and AT phases (*P* < 0.05) (Figures 2A,C), although the proportions of PD-1^+^ bulk CD8^+^ T cells did not differ from those in the other phases (Figure 2D). We also examined the expression of CD127, a representative memory marker (34). CD127 is a cell surface marker that identifies CD8^+^ T cells that will become memory CD8^+^ T cells, in both the mouse models and in humans with acute resolving viral infection (35). In chronic HBV infection, a recent study demonstrated that the CD127^+^PD1^+^ subset is a memory-like population in chronic viral infection, and that the frequency of this subset clearly correlated with the expansion capacity of HBV core_18−27_-specific CD8^+^ T cells in chronic HBV infection (34). In our analyses, we observed no significant changes in the proportions of CD127^+^ HBV-specific CD8^+^ T cells among different infection stages (data not shown). However, when we calculated the proportions of CD127^low^PD-1^+^ CD8^+^ cells, the proportion of CD127^low^PD-1^+^ HBV core_18−27_-specific CD8^+^ T cells was significantly lower in patients in the IT phase than in those in the AT phase (Figures 2A,C).

Furthermore, we performed additional analyses with pentamer^+^ CD8^+^ T cells for Tim-3 and Lag-3 as surface markers, and T-bet and Eomes as transcription factors. The gating strategy is presented in Supplementary Figure 5. Unfortunately, we could not perform these analyses in all the enrolled patients because of limited sample availability. For HBV core_18−27_ specific CD8^+^ T cells, we confirmed that pentamer-specific CD8^+^ T cells showed significantly higher levels of Tim-3 expression compared to in bulk CD8^+^ T cells in most enrolled patients, and the levels were higher in IA and AT patients than in IT patients; however, the difference was not significant because of the small number of samples (Supplementary Figure 6). Lag-3 was not readily detected in our samples (Supplementary Figure 6). In general, high expression of Eomes accompanied by low levels of T-bet has been linked to T cell exhaustion (36). In our cohort, T-bet and Eomes expression was examined in selected patients because of limited sample availability, and no significant differences were observed in T-bet and Eomes expression of HBV core_18−27_-specific CD8^+^ T cells among IT, IA, and AT patients (Supplementary Figure 6).

### Proportions of PD-1^+^ CD8^+^ T Cells and CD127^low^PD-1^+^ CD8^+^ T Cells in HBV-Specific CD8^+^ T Cells Correlated With Patient age

To investigate potential contributing factors associated with the surface expression of PD-1 in HBV-specific CD8^+^ T cells, we performed correlation and regression analyses. Factors such as the level of HBV DNA in the IT and IA phases, ALT level in the IT, IA, and AT phases, and duration of AT in the AT phase, were not correlated with the PD-1 level in HBV-specific CD8^+^ T cells (data not shown). The proportions of PD-1^+^ cells and CD127^low^PD-1^+^ CD8^+^ T cells in HBV core_18−27_-specific CD8^+^ T cells were positively correlated with patient age (Figures 2E,F). The proportion of PD-1^+^ cells in bulk CD8^+^ cells also correlated with patient age (Figure 2G), although the correlation coefficient was higher in HBV-specific CD8^+^ T cells. The number of SFUs in ELISpot or the proportion of pentamer^+^ cells was not significantly associated with patient age (data not shown). Overall, these data suggest that HBV-specific CD8^+^ T cells in the IT phase expressed low levels of PD-1, and that the PD-1^+^ cell population increased as the patients aged.

### Virus-Specific T Cells From Patients With Chronic HBV Infection Show Defective IFN-γ Production *ex vivo*

**Figure 3:**
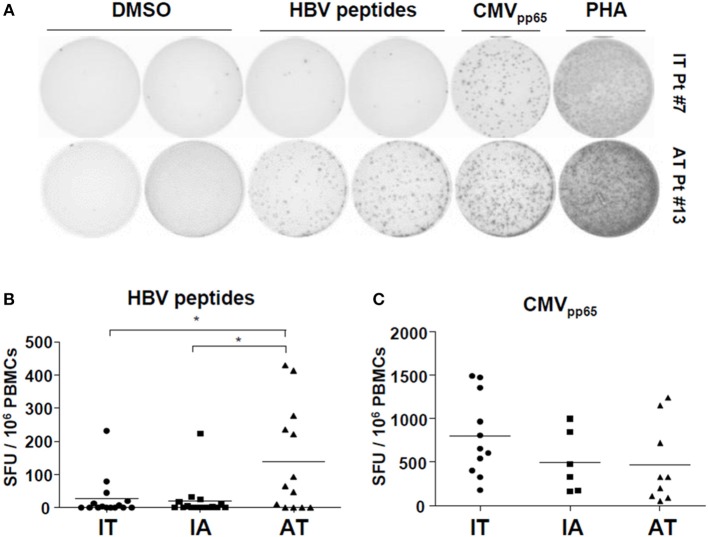
Defective *ex vivo* IFN-γ production by HBV-specific T cells in patients with chronic HBV infection. **(A,B)** Direct *ex vivo* ELISpot assay using an HBV peptide mixture was performed on PBMCs from the patient cohort (IT: *n* = 15, IA: *n* = 14, AT: *n* = 13). PBMCs were stimulated with the peptide mixture at a final concentration of 1 μg/mL for 24 h at 37°C. **(A)** Representative ELISpot result. **(B)** Results of *ex vivo* ELISpot using the peptide mixture in each group in the cohort. *P < 0.05. **(C)** Results of *ex vivo* ELISpot using CMV_pp65_ OLPs in each group in the cohort. AT, antiviral treatment; CMV, cytomegalovirus; ELISpot, enzyme-linked immunospot; HBV, hepatitis B virus; IA, immune-active; IFN, interferon; IT, immune-tolerant; OLPs, overlapping peptides; PBMC, peripheral blood mononuclear cell; SFU, spot-forming unit.

Next, we performed *ex vivo* IFN-γ ELISpot assays using PBMCs and a mixture of HBV peptides (Supplementary Table 2). Consistent with previous reports (13, 24), *ex vivo* ELISpot did not detect robust IFN-γ responses within the IT and IA groups, although some patients on AT with very low HBV DNA levels had PBMCs showing notable *ex vivo* IFN-γ production (Figures 3A,B). The AT group had a significantly larger number of SFUs than the IT and IA groups (*P* < 0.05) (Figure 3B and Supplementary Figure 7). There were no differences in the number of SFUs among patients with different antiviral agents. We performed ELISpot using CMV_pp65_ OLPs to exclude general activation of non-HBV-specific T-cell responses and observed no difference in the CMV-specific T cell response among the three groups (Figure 3C). *Ex vivo* ELISPOT using OLPs from HBsAg and HBcAg also revealed poor IFN-γ responses in selected patients within the IT and IA groups (Supplementary Figure 8). Together, these findings suggest that patients with chronic HBV infection are defective in *ex vivo* IFN-γ production after stimulation with HBV peptides, which can be partly restored by treatment-induced viral suppression.

### Multimer-Stained CD8^+^ T Cells in the IT Phase Show Defective IFN-γ Production *ex vivo* After Peptide Stimulation

**Figure 4:**
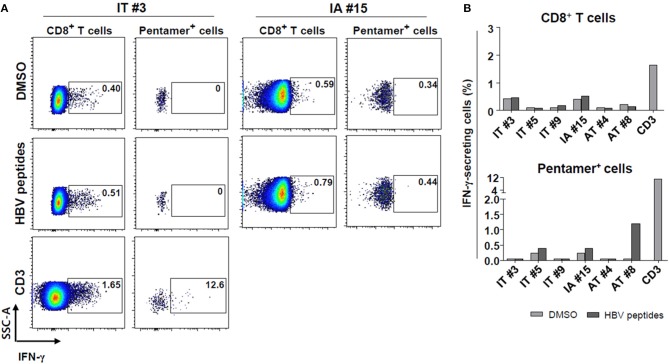
Defective IFN-γ production *ex vivo* by HBV core_18−27_-pentamer-stained CD8^+^ T cells in the IT phase after peptide stimulation. **(A,B)** Secretion of IFN-γ by bulk and pentamer^+^ CD8^+^ T cells as examined using the cytokine secretion assays. **(A)** Representative dotplot describing the proportion of IFN-γ-secreting cells after peptide stimulation. **(B)** Proportion of IFN-γ-secreting cells after HBV peptide stimulation in each patient sample analyzed. Anti-CD3 antibody was used for a positive control. AT, antiviral treatment; DMSO, dimethyl sulfoxide; IA, immune-active; IFN, interferon; IT, immune-tolerant.

Subsequently, *ex vivo* cytokine secretion assays using a capture antibody for IFN-γ were conducted to confirm the defective secretion of IFN-γ in HBV core_18−27_ pentamer-stained cells from IT patients (Figures 4A,B). Initially, we attempted to combine intracellular cytokine staining and pentamer staining procedures to determine whether pentamer^+^ cells in the IT phase have antiviral functions (although ELISpot did not show significant results). However, combining intracellular cytokine staining and direct pentamer staining shows some limitations (29). It is known that after stimulation, T cell receptors may be downregulated or there may be stimulation from the tetramer itself (29). Therefore, we instead performed an *ex vivo* secretion assay. Pentamer-stained HBV-specific CD8^+^ T cells were defective in IFN-γ secretion in patients in the IT group, although some patients in AT group showed *ex vivo* IFN-γ secretion after peptide stimulation (Figures 4A,B). CD3 stimulation of PBMCs from patients in the IT group led to robust IFN-γ secretion in both of bulk CD8^+^ T cells and pentamer^+^ cells (Figures 4A,B).

### HBV-Specific CD8^+^ T Cells From Patients in the IT Phase Can Produce IFN-γ After *in vitro* Culture With HBV Peptides

**Figure 5:**
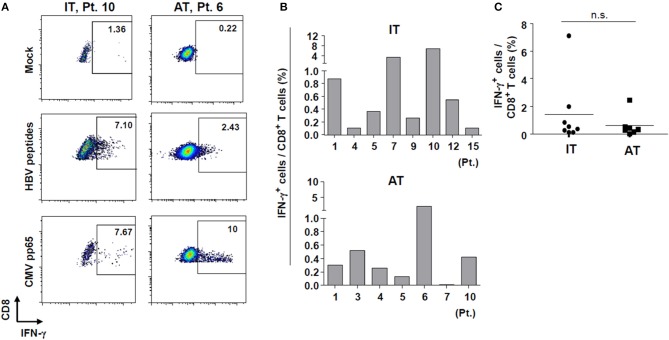
IFN-γ production by HBV-specific T cells after *in vitro* culture with HBV peptides. **(A)** Representative dotplot describing the proportion of IFN-γ-producing CD8^+^ T cells after *in vitro* culture with HBV peptides. **(B,C)** Proportion of IFN-γ-producing CD8^+^ T cells after *in vitro* culture with HBV peptides in each patient sample analyzed. AT, antiviral treatment; CMV, cytomegalovirus; HBV, hepatitis B virus; IFN, interferon; IT, immune-tolerant; n.s., not significant.

Next, we performed a 10-day *in vitro* culture of PBMCs with an HBV peptide mixture (Supplementary Table 2) and IL-2. We performed *in vitro* expansion of HBV-specific CD8^+^ T cells from selected patients enrolled in the study because of limited sample availability from some patients. After 10 days of culture, the percentages of IFN-γ-producing CD8^+^ T cells from some patients in the IT group were significantly increased (Figures 5A,B) despite the defective IFN-γ production seen in the *ex vivo* ELISpot results (Figure 3B). Cells from one patient (Patient #10) in the IT group, which showed a minimal response in the *ex vivo* ELISpot, produced IFN-γ robustly after 10-day culture (7.1% IFN-γ-producing cells among all CD8^+^ T cells). There were no significant differences in the frequency of IFN-γ-producing CD8^+^ T cells between IT and AT groups (Figure 5C). These data demonstrate that HBV-specific CD8^+^ T cells in patients considered to be in the IT phase may be activated and secrete IFN-γ when appropriately stimulated.

## Discussion

In this study, we confirmed the presence of HBV-specific CD8^+^ T cells and low proportion of PD-1^+^ cells in patients considered as being in the IT phase. Although HBV-specific T cells in the IT phase do not readily produce IFN-γ *ex vivo*, they can be activated and produce IFN-γ after persistent *in vitro* stimulation. Furthermore, the proportion of PD-1 expression in HBV-specific CD8^+^ T cells is lower in this phase than in subsequent phases despite the high viral load. Our data suggest that appropriate stimulation can enhance the effector function of HBV-specific CD8^+^ T cells in patients considered as IT, and future immunomodulatory approaches should target these patients.

In East Asia, chronic HBV infection is typically established in early childhood, resulting in many young patients with CHB in the IT phase. Traditionally, the IT phase has been associated with a lack of disease activity. Therefore, the international guideline did not recommend treatment of patients in the IT phase with antiviral agents (22). However, a recent study demonstrated that integration of HBV-DNA and clonal expansion of hepatocytes occurred in patients considered IT (14). Because the authors detected HBV-specific T-cell responses in PBMCs, they suggested that clonal expansion of hepatocytes may result from T-cell-mediated killing of hepatocytes (14). In agreement with the report, our results further demonstrate that HBV-specific CD8^+^ T cells are composed of a low proportion of PD-1^+^ cells *ex vivo* in these patients and the cells can be activated by appropriate stimuli as well as produce IFN-γ. Direct *ex vivo* functional analysis of HBV-specific T cells without *in vitro* expansion is performed by measuring cytokine secretion or cell proliferation upon *in vitro* stimulation with HBV antigens for a few hours. This better represents the physiological nature of immune responses.

Immune tolerance to specific pathogens encompasses both deletional and functional tolerance. Deletional T-cell tolerance mainly affects T cells with high affinity to their cognate antigen (37). Functional tolerance is caused by silencing of T cell activation in an antigen-dependent manner by cell intrinsic mechanisms, interacting with inhibitory molecules on target cells, or inhibitory molecules or regulatory cells around the T cell (37). These mechanisms appear to be maximally exploited by HBV. Previous reports demonstrated that the number of effector T cells detected by *ex vivo* ELISpot in chronic HBV infection was exceptionally low (13, 14, 24, 37–39). This may have been caused by deletion of HBV-specific T cells or functional unresponsiveness. Therefore, detecting the HBV-specific T cell response in the IT phase of HBV infection *ex vivo* is difficult using ELISpot. As an alternative, *in vitro* culture of PBMCs for 10 days was performed before the various analyses to detect T cell responses. In this study, we did not detect *ex vivo* ELISpot responses in patients considered as immune tolerant. However, although the numbers were small in selected samples, we detected HBV-specific CD8^+^ T cells *ex vivo* using multimer staining, suggesting that not all HBV-specific CD8^+^ T cells had been eliminated. Consistent with our data, Chinese groups described the *ex vivo* multimer detection (HBV core_18−27_ pentamer) of HBV-specific CD8^+^ T cells (30, 40–42). In Korea and China, most patients with HBV are infected with genotype C HBV (19), indicating that viral and host factors from different regions worldwide influence the detectability of HBV-specific CD8^+^ T cells using the HBV core pentamer.

A previous study demonstrated that the single amino acid alteration of valine to isoleucine at the position of the HBV core 27 amino acids may reduce the binding affinity to HLA-A^*^02 by 10-fold (31). This may result in an insufficient CD8^+^ T cell response to the FLPSDFFPSI epitope or inefficient deletion of CD8^+^ T cell precursors responsive to FLPSDFFPSI in chronic infection (31). However, a very recent study showed that sequence variations in the core_18−27_ region may not account for the epitope-specific CD8^+^ T cell phenotypes (32). Despite the replacement of valine to isoleucine at the core 27 amino acid position, the responses to the core_18−27_ epitope displayed the most homogeneous phenotypic profiles, with strong expression of both PD-1 and CD127 in nearly all chronic patients (32). This suggests that the sequence data do not completely explain the phenotypic differences observed between HBV-specific CD8^+^ T cell responses, and that CD8^+^ T cell responses to the HBV core_18−27_ epitope in patients with FLPSDFFPSI sequence variation are a useful marker of the CD8^+^ T cell response in chronic HBV infection.

PD-1 expression is known as the classical hallmark of exhausted T cells (2). However, Rivino et al. recently demonstrated that the frequency of HBV-specific T cell responses was higher in patients without flares after stopping antiviral therapy, and these cells were most commonly found in the PD-1^+^ T cell compartment (33). They found that these PD-1^+^ T cell populations were functional, at least in terms of their proliferative capacity and ability to produce IFN-γ (33). Similarly, recent studies showed that patients with partial immune control of HBV infection display higher levels of intrahepatic PD-1^+^ CD39^+^ tissue-resident CD8^+^ T cells with the capacity to mount robust cytokine responses (43). Based on this information, *ex vivo* PD-1 expression on the surface of HBV-specific CD8^+^ T cells may not be associated with defective production of IFN-γ. Our data agree with previous reports showing that patients under AT express relatively higher levels of PD-1 in their HBV-specific CD8^+^ T cells, although their *ex vivo* IFN-γ production is higher than that of patients considered IT. The age-dependent increase in *ex vivo* PD-1 expression in HBV-specific CD8^+^ T cells suggests that PD-1 is associated with the duration of antigen exposure in patients with chronic HBV infection (44). A recent report also demonstrated that expression of T cell immunoreceptor with the Ig and ITIM domains (TIGIT), another immune checkpoint molecule, increases with age on hepatic CD8^+^ T cells in HBsAg-transgenic mice whose adaptive immunity is tolerant to HBsAg (45).

Our study had some limitations. First, most of the data presented in this study are from the multimer analysis with an HLA-A^*^02 multimer (HBV core_18−27_). Recently, two independent groups demonstrated that phenotype of HBV-specific T cells may differ when the targeted epitope is changed (32, 34, 36). We also performed analyses with an HBV polymerase_455−463_ pentamer, but only in selected patients because of sample availability. Moreover, we could not perform multimer analyses to detect other epitope-specific CD8^+^ T cells. Second, we performed *ex vivo* functional analysis only by quantifying IFN-γ production. More detailed analyses were not performed because of limited sample availability. Finally, also because of sample limitations, we could not evaluate multiple exhaustion markers in all samples, although we measured Tim-3 and Lag-3 in some of the samples and observed significantly higher levels of Tim-3 expression in most enrolled patients compared to in bulk CD8^+^ T cells. These results must be validated in larger-scale studies.

In conclusion, we confirmed the presence of HBV-specific CD8^+^ T cells and low proportion of PD-1^+^ cells in patients considered as being in the IT phase. HBV-specific CD8^+^ T cells were not activated *ex vivo* but could be activated in *in vitro* culture in the IT phase. The age-dependent increase in *ex vivo* PD-1 expression in HBV-specific CD8^+^ T cells indicates that the proportion of PD-1 expression in HBV-specific CD8^+^ T cells was lower in this phase than in the following phases. Our data suggest that future immunomodulatory approaches should target IT patients because their virus-specific CD8^+^ T cells are not exhausted or tolerant. A longitudinal study is needed to confirm the changes in the phenotype and function of *ex vivo* HBV-specific T cells in patients with chronic HBV infection.

## Ethics Statement

Informed consent in writing was obtained from all patients. The present study was conducted according to the Declaration of Helsinki principles and was approved by the Institutional Review Boards (Seoul St. Mary's Hospital, KC16 MISI0714).

## Author Contributions

PS and SY: study design, data collection, data analysis, data interpretation, manuscript writing, and manuscript approval. DP: data collection, data analysis, data interpretation, and manuscript writing. J-HK, JH, EL, GL, and HN: data collection. JJ, SB, JC, E-CS, and S-HP: data interpretation and manuscript approval.

### Conflict of Interest Statement

The authors declare that the research was conducted in the absence of any commercial or financial relationships that could be construed as a potential conflict of interest.
