# Supplementary material for: Ex vivo Detection and Characterization of Hepatitis B Virus-Specific CD8+ T Cells in Patients Considered Immune Tolerant
Source: Front Immunol. 2019 Jun 6;10:1319. doi: 10.3389/fimmu.2019.01319 (PMC6563765; doi:10.3389/fimmu.2019.01319)
Supplement: Supplementary file 1 [file Table_1.DOCX]

**Supplementary Experimental Procedures**

**Supplementary Table 1. Primer sequences used in this study**

|  | **Sequence** |
| --- | --- |
| **1st HBV core PCR – Forward** | 5’-TGTCAACGACCGACCTTGAGG-3’ |
| **1st HBV core PCR – Reverse** | 5’-TGTAGCTCTTGTTCCCAA-3’ |
| **2nd HBV core PCR – Forward** | 5’-AGGCTGTAGGCATAAATTGGT-3’ |
| **2nd HBV core PCR – Reverse** | 5’-TTCCCACCTTATGAGTCCAAG-3’ |

**Supplementary Table 2. Sequences of minimal epitopes used in this study**

| Peptide | Sequence | Epitope source | HLA Allele Restriction |
| --- | --- | --- | --- |
| 1 | FLLSLGIHL | HBV polymerase _573-581_ | A^*^02:01 |
| 2 | FLLTRILTI | HBV envelope _183-191_ | A^*^02:01 |
| 3 | FLPSDFFPSI | HBV core _18-27 (subtype ADR4)_ | A^*^02:01 |
| 4 | FLPSDFFPSV | HBV core antigen _18-27_ | A^*^02:01 |
| 5 | GLSPTVWLSV | HBV surface antigen _185-194_ | A^*^02:01 |
| 6 | WLSLLVPFV | HBV surface antigen _172-181_ | A^*^02:01 |


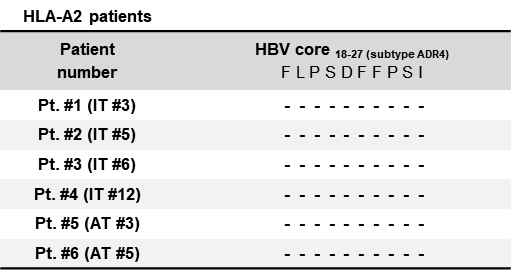
**Supplementary Table 3. Viral sequences of HBV core_18-27_ region**
