# Supplementary material for: Ex vivo Detection and Characterization of Hepatitis B Virus-Specific CD8+ T Cells in Patients Considered Immune Tolerant
Source: Front Immunol. 2019 Jun 6;10:1319. doi: 10.3389/fimmu.2019.01319 (PMC6563765; doi:10.3389/fimmu.2019.01319)

Supplementary Figure 1. Comparison of HLA-A*02 pentamer corresponding to HBV core$_{18-27}$ FLPSDFFPSV and HBV core$_{18-27}$ FLPSDFFPSI

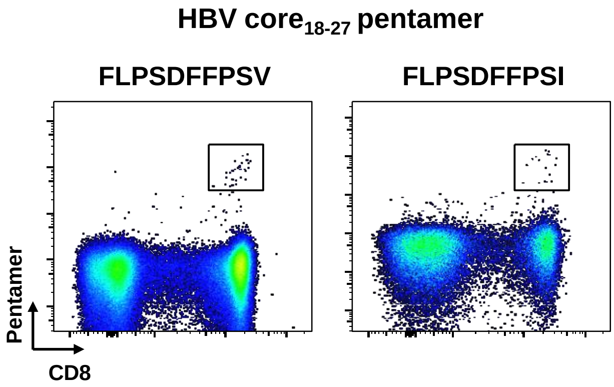

Supplementary Figure 2. Proportion of HBV core$_{18-27}$-specific CD8^{+} T cells as determined using flow cytometry in normal controls and patients in the IT, IA, and AT groups

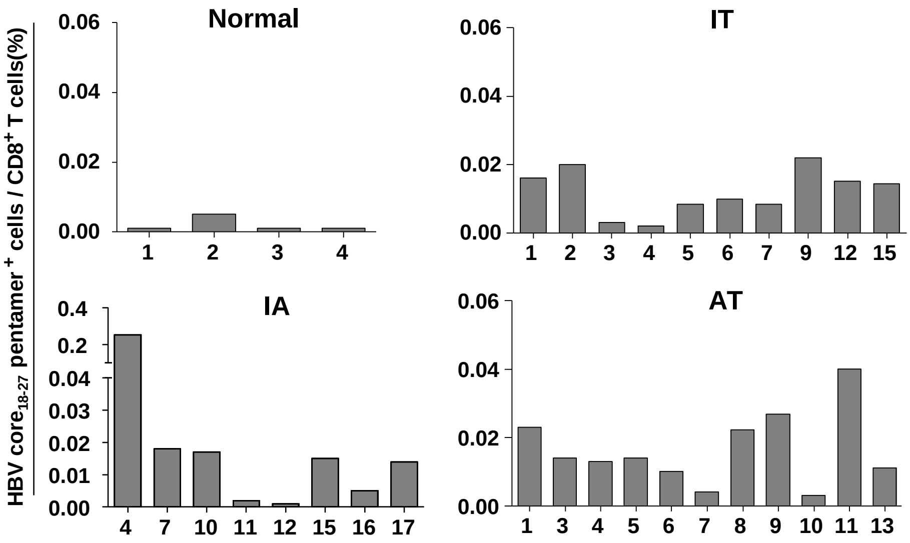

Supplementary Figure 3. Detection of HBV pol$_{455\text{-}463}$-specific CD8$^+$ T cells in selected patients with acute HBV infection

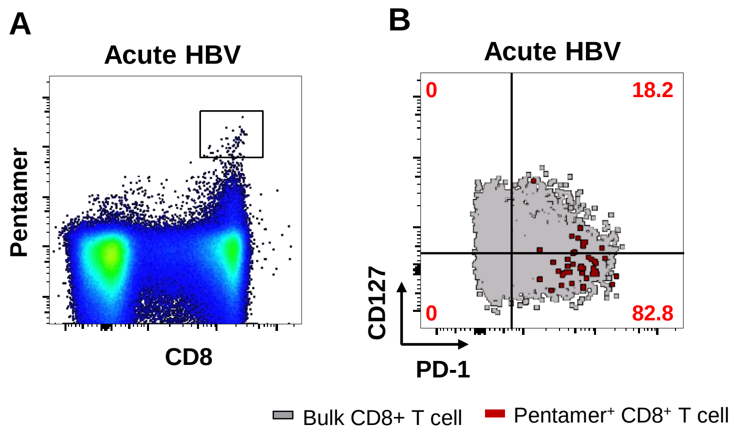

Supplementary Figure 4. Gating strategy of surface markers (CCR7 and CD45RA) in pentamer$^+$CD8$^+$ T cells

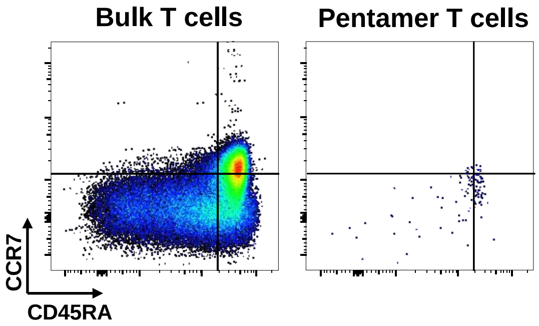

Supplementary Figure 5. Gating strategy of surface and intracellular markers in pentamer$^+$CD8$^+$ T cells

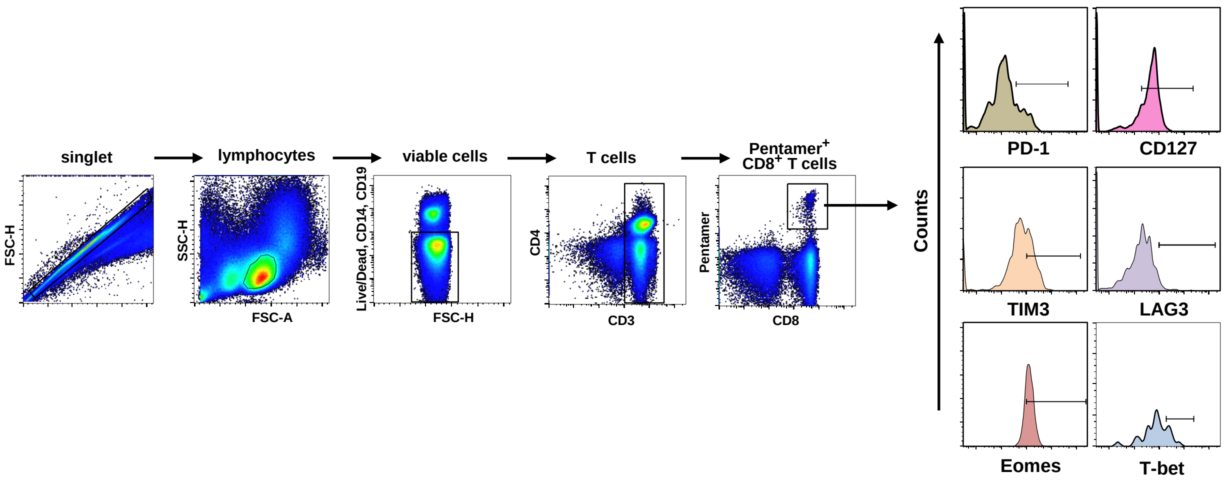

Supplementary Figure 6. Frequency of surface and intracellular markers in pentamer$^+$CD8$^+$ T cells in selected patients with chronic HBV infection

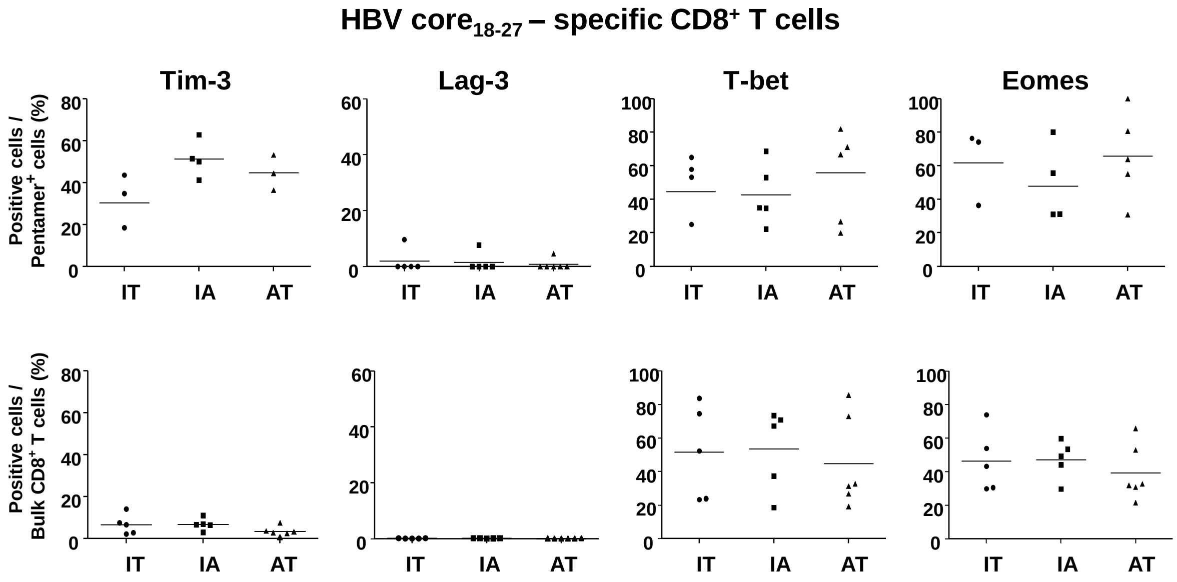

Supplementary Figure 7. Defective *ex vivo* IFN-γ production by HBV-specific T cells when stimulated with the peptide mixture in patients with chronic HBV infection

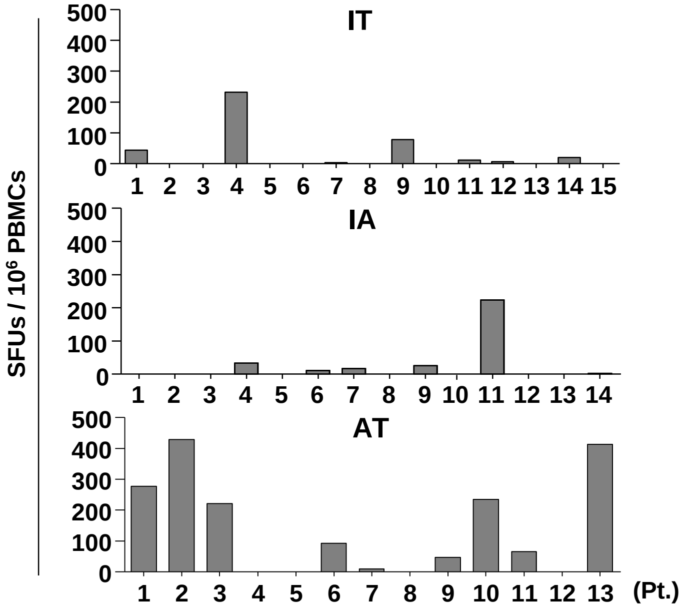

Supplementary Figure 8. Defective *ex vivo* IFN-γ production by HBV-specific T cells when stimulated with overlapping peptides in patients with chronic HBV infection

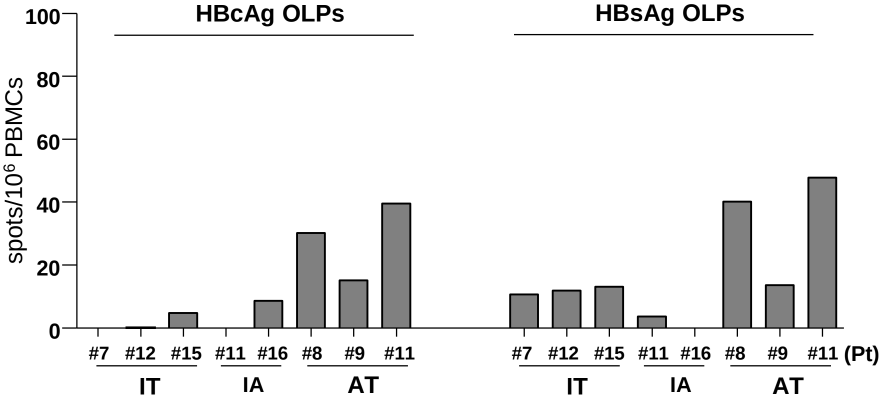

Supplement: Supplementary file 2 [file Presentation_1.pdf]
